# Anti-bacterial, free radical scavenging activity and cytotoxicity of acetone extracts of *Grewia flava*

**DOI:** 10.4314/ahs.v17i3.22

**Published:** 2017-09

**Authors:** Stella Makgabo Lamola, Jean Paul Dzoyem, Francien Botha, Candice van Wyk

**Affiliations:** 1 Phytomedicine Programme, Department of Paraclinical Sciences, Faculty of Veterinary Science, University of Pretoria, Private Bag X04, Onderstepoort 0110, Pretoria, South Africa; 2 Department of Biochemistry, Faculty of Science, University of Dschang, P.O. Box 67, Dschang, Cameroon; 3 Department of Community Dentistry, Faculty ofHealth Sciences, University of Pretoria, Pretoria, P. O. Box 1266, Pretoria 0001, South Africa

**Keywords:** Extracts, anti-microbial, cytotoxicity, enteric pathogens, *Grewia flava*

## Abstract

**Background:**

Bacterial infections of the gastrointestinal tract (GIT) cause vomiting, diarrhoea and even systemic disease. There is a need for the development of natural products into alternative and safer medicines.

**Objectives:**

This study evaluated the anti-microbial activity of extracts prepared from berries, leaves, bark and roots of the edible plant *Grewia flava*.

**Methods:**

The anti-bacterial activity was evaluated by the broth microdilution method. Anti-oxidant activity of the most active extracts was performed by 2, 2-diphenyl-1-picrylhydrazyl (DPPH) assay. The cytotoxicity of the extracts was determined using the 3-(4,5-dimethylthiazol-2-yl)-2,5-diphenyltetrazolium bromide (MTT) assay.

**Results:**

The acetone extracts of the leaves and roots showed the best activity with MIC values as low as 0.03 mg/mL against *Staphylococcus aureus* and *Salmonella typhimurium* and 0.07 mg/mL against *Bacillus cereus, Escherichia coli* and *Staphylococcus aureus*. Quantitative analysis of the scavenging ability showed that acetone extracts exhibited good free radical scavenging activity in a dose-dependent manner. The berries extract had the highest LC_50_ (lowest toxicity) of 551.68 68 µg/mL.

**Conclusion:**

Acetone extract of leaves and roots of *Grewia flava* contain anti-microbial and anti-oxidant compounds and could therefore be used as a natural product with little toxicity to host cells.

## Introduction

For centuries the indigenous people of South Africa have relied on herbal medicine for all their primary health care, and it is estimated that millions of South Africans still use traditional remedies from as many as 700 indigenous plant species^1^. The use of medicinal plants in the world contributes significantly to primary health care and traditional medicines are part of the cultural and religious beliefs, they are easily available and affordable to rural people^2^,^3^. Therefore the level of sanitation, hygiene and living conditions in most rural areas are not comparable to those of urban areas^4^. There are numerous bacteria species that can infect the digestive system, such as *Salmonella* spp., *Escherichia coli, Staphylococcus aureus*^5^. The search for and development of new anti-biotics that will target resistant bacterial strains are needed. Therefore, the screening of plant extracts for antibacterial compounds is very important and relevant^6^. Anti-microbial substances from natural sources like plants have been investigated to achieve higher levels of food safety^7^. Medicinal plants are assumed to be non-toxic and regarded safe due to their natural origin and the long use in traditional medicine to treat various forms of diseases^8^,^9^. Scientific studies on efficacy and safety of some of the medicinal plants indicated that there are many phytochemicals that have cytotoxicity, genotocixity and carcinogenic effects when used chronically^10^.

The genus *Grewia* is from the family *Malvaceae* and a major angiosperm group (flowering plants) with approximately 400 species of flowering plants, shrubs, and trees that are widely distributed in sub-tropical and tropical regions^11^,^12^. A number of species of the genus *Grewia* have been reported to be used as medicinal agents to treat several diseases such as skin diseases, hypertension, ulcers and diarrhoea, and compounds were isolated from various species of the *Grewia*^13^. *Grewia flava* DC. is commonly known as the “brandy bush” or “velvet raisin” (English), “*fluweelrosyntjie/wilderosyntjie*” (Afrikaans) and “*moretlwa*” (Tswana and Sepedi)^14^. The *Grewia flava* fruits are mashed, soaked for a while in water and eaten as porridge by the San (Bushmen). The beer prepared from the berries is called “Khadi”^14^. There is a diverse possibility that the plant involved has ethno-medicinal familiarity to the indigenous people and the potential pharmacological interactions are an area in serious need of in-depth study^15^. To the best of the author's knowledge, no study has been focused on the pharmacological activity of *Grewia flava*. The aim of the study was to analyze the anti-microbial, the free radical scavenging activity and the cytotoxicity of extracts from the edible plant *Grewia flava*.

## Materials and methods

### Plant materials

Berries, leaves, barks and roots from the edible plant *Grewia flava* were collected from an open field at Moletjie Ga-Phago near Lonsdale, Polokwane, Limpopo (Grid 2329CA 23°00′, 29°00″ E). The plant was identified and authenticated by Elsa van Wyk from the University of Pretoria and a voucher specimen, PRU 119004, is maintained at the HGWJ Schweickerdt Herbarium of the Department of Plant Science, University of Pretoria. Plant material was dried at room temperature in the shade and ground to powder using a Kika-werk M20, bench top grinder. Powdered materials were stored in closed honey jars at room temperature until use.

### Extraction

One gram of each plant part was macerated in 10 mL of acetone, methanol, acetyl acetate and water in polyester centrifuge tubes. The tube was vigorously shaken for 30 min on an orbital shaker, then centrifuged at 4000 x g for 10 min and the supernatant was filtered using Whatman No.1 filter paper before being transferred into pre-weighed glass containers. This was repeated thrice and solvent was removed by evaporation under a stream of air in a fume hood at room temperature to produce the dried extract.

### TLC fingerprinting

TLC-vanillin method of Kotze and Eloff was used^16^. The dried extracts were weighed and the mass of the dried residues recorded. Ten milligrams (10 mg) of the dried extract residue was weighed and a 10 mg/mL concentration extract solution was prepared. Each extract (10 µL) was loaded on the base of the thin layer chromatography silica gel 60 F254 aluminium backed plate (Merck). Different mobile systems were used for separation of the compounds namely: Ethyl acetate: methanol: water (EMW, polar system); chloroform: ethyl acetate: formic acid (CEF, intermediate system); and benzene: ethanol: acetic acid (BEA, non-polar system). The plates were sprayed with vanillin in sulphuric acid (0.1 g vanillin in 28 ml methanol and 1 ml H_2_SO_4_) for visualising different compounds in the extracts.

### Anti-microbial activity

The four bacterial strains used included two Gram-positive bacteria (*Staphylococcus aureus* ATCC 29213, *Bacillus cereus* ATCC14579) and two Gram-negative bacteria (*Escherichia coli* ATCC 25922 and *Salmonella typhimurium* ATCC 14028). The minimum inhibitory concentration (MIC) was determined as previously described^17^.

### Anti-oxidant activity

#### Qualitative analysis of the anti-oxidant activity

The TLC-DPPH method and TLC-vanillin method of Masoko and Eloff was used^18^. The dried extracts were weighed and the mass of the dried residues recorded. Ten milligrams (10 mg) of the dried extract residue was weighed and a 10 mg/mL concentration extract solution was prepared. Each extract (10 µL) was loaded on the base of the thin layer chromatography silica gel 60 F254 aluminium backed plate (Merck). Different mobile systems were used for separation of the compounds namely: Ethyl acetate: methanol: water (EMW, polar system); chloroform: ethyl acetate: formic acid (CEF, intermediate system); and benzene: ethanol: acetic acid (BEA, non-polar system). The plates were sprayed with 0.2% DPPH in methanol to determine the free radical scavenging activity. Other plates were sprayed with vanillin in sulphuric acid (0.1 g vanillin in 28 ml methanol and 1 ml H_2_SO_4_) for visualising different compounds in the extracts.

#### DPPH radical scavenging activity

2,2-diphenyl-1-picrylhydrazyl (DPPH) radical-scavenging activity was determined using the method proposed by Brand-Williams^19^. One hundred and sixty microlitres of the methanolic solution of DPPH (0.04 mg/mL,) was added to 40 µl ascorbic acid and Trolox at concentrations of 1.0–200 µg/mL (positive controls), and different concentrations of crude extracts (3.9–500 µg/mL). After 30 minutes the absorbance was measured at 517 nm using a Biotek microplate reader. Ascorbic acid was used as positive control, methanol as negative control and extract without DPPH as blank. Scavenging capacity (%) = 100-[(absorbance of sample - absorbance of sample blank)×100/(absorbance of control)-(absorbance of control blank)]. The IC_50_ values were calculated from the graph plotted as inhibition percentage against the concentration.

### Cytotoxicity

Cytotoxicity of extracts was evaluated against Vero cells using the 3-(4,5-dimethylthiazol-2-yl)-2,5-diphenyltetrazolium bromide (MTT) assay as previously described^17^. Cells were seeded at a density of 1 x 10^5^ cells/ml (100 µl) in 96-well microtitre plates and incubated at 37°C and 5% CO_2_ in a humidified environment. After 24h incubation, extracts (100 µl) at varying final concentrations were added to the wells containing cells. Doxorubicin was used as a positive reference. A suitable blank control with equivalent concentrations of acetone was also included and the plates were further incubated for 48h in a CO_2_ incubator. Thereafter, the medium in each well was aspirated from the cells, which were then washed with PBS, and finally fresh medium (200 µl) was added to each well. Then, 30 µl of MTT (5 mg/ml in PBS) was added to each well and the plates were incubated at 37°C for 4h. The medium was aspirated from the wells and DMSO was added to solubilize the formed formazan crystals. The absorbance was measured on a BioTek Synergy microplate reader at 570 nm. Cell growth inhibition for each extract was expressed in terms of LC^50^ values, defined as the concentration that caused 50% of inhibition of cell viability. The selectivity index (SI) values were calculated by dividing cytotoxicity LC^50^ values by the MIC values (SI = LC^50^/MIC). Tests were carried out in quadruplicate and each experiment was repeated thrice.

### Statistical analysis

All experiments were conducted thrice in triplicates and values expressed as mean ± standard deviation (SD). Statistical analysis was performed by using one way ANOVA and results were compared using the Student's t-Test at a 5% significance level.

## Results and discussion

### Anti-microbial activity

The anti-bacterial activity of the *Grewia flava* plant extracts parts is shown in Table 1. The results showed that the activity of the extracts against the four enteric pathogens varied from weak to significant with MIC values ranged from >2.5 to 0.03 mg/mL.The acetone extracts of the leaves and roots had better activity with MIC values as low as 0.03 mg/mL against *S. aureus* and *S. typhimurium* and 0.07 mg/mL against *B. cereus, E. coli* and *S. aureus*. Acetone was previously reported to be the best extractant of anti-microbial compounds in addition to its low toxicity to pathogens^20^. Methanol extracts also had significant activity with the lowest inhibition at a concentration of 0.07 mg/mL from the leaves, bark and roots against *E. coli, B. cereus* and *S. aureus*. Acetyl acetate root extract had an effective activity against *S. aueus* and *E. coli* with a minimal concentration of 0.07 mg/mL. Water extracts were not effective against the enteric pathogens.

**Table 1:** Minimal inhibitory concentrations (mg/mL) of the berries, leaves, bark and roots extracts of *Grewia flava* against four enteric pathogens.

Plant parts		MIC (mg/mL)			
		
	Solvents	*Sa*	*Bc*	*Ec*	*ST*
**Berries**	Acetone	0.62	1.25	0.31	1.25
	Methanol	>2.5	>2.5	1.25	>2.5
	Acetyl acetate	0.15	0.15	0.31	0.31
	Water	0.31	1.25	0.15	0.62
**Leaves**	Acetone	**0.03**	**0.07**	**0.07**	**0.03**
	Methanol	**0.07**	0.62	**0.07**	0.15
	Acetyl acetate	0.62	1.25	0.31	1.25
	Water	0.31	>2.5	0.31	1.25
**Barks**	Acetone	**0.07**	0.31	0.15	0.62
	Methanol	0.31	0.15	0.31	0.31
	Acetyl acetate	0.62	1.25	0.15	1.25
	Water	>2.5	>2.5	1.25	>2.5
**Roots**	Acetone	**0.03**	**0.07**	**0.07**	0.62
	Methanol	**0.07**	0.31	**0.07**	0.15
	Acetyl acetate	0.15	0.31	0.62	0.31
	Water	0.31	>2.5	1.25	0.62
**Gentamicin** **(µg/mL)**		0.2	0.8	0.4	1.56

*BC: Bacillus cereus, EC: Escherichia coli, ST: Staphylococcus aureus, ST: Salmonella typhimurium.* in bold are MIC values < 0.1 mg/mL.

To the best of the author's knowledge, this is the first report on the anti-bacterial activity of extracts from *Grewia flava* species. However, several previous studies reported the antibacterial activity of extracts from the genus *Grewia*^21^. For instance, Gupta et al.^22^ reported the activity of different extracts of *G. asiatica* against four Gram positive (*Bacillus subtilis, B. cereus, Staphylococcus aureus, Enterococcus faecalis*) and five Gram negative bacteria (*Escherichia coli, Listeria monocytogeneses, Salmonella typhimurium, Shigella flexneri* and *Pseudomonas aerugenosa*).

Although the methanol extracts were also effective against the enteric pathogens, the acetone extracts were the most active and were selected for further studies.

### TLC fingerprinting

**Figure 1:**
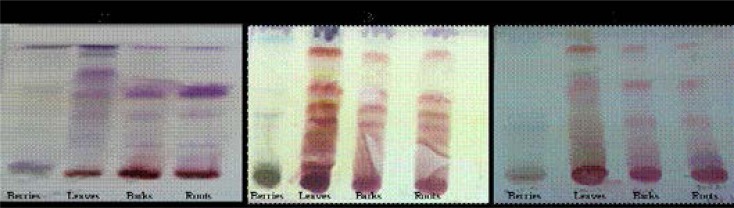
TLC chromatogram of the berries, leaves, bark and roots extracts developed in the BEA system (A) in the EMW system (B) and in the CEF system (C) and sprayed with vanillin--H_2_SO_4_.

In order to observe the major compounds group within the most active extracts, qualitative phytochemical analysis using TLC fingerprinting was developed in three different systems: a non-polar system (BEA), an intermediate system (CEF) and a polar system (EMW). Qualitative screening of acetone extracts of the *Grewia flava* plant parts was performed to obtain thin layer chromatography (TLC) fingerprints of each investigated extract. Results are presented in Figure 1. The chromatogram reveals a mixture of compounds where the system separated the non-polar compounds finely, which exhibited different colours when reacting to the Vanillin/H_2_SO_4_ spray reagent. The classes of compounds extracted include the terpenoids, which are purple or bluish purple^23^. The EMW system, which is a highly polar system, was used to separate the water extracts, because water is a highly polar solvent, most of the compounds extracted were polar to highly polar compounds. Some secondary metabolites and isolated compounds present in *Grewia* species include pelargonidin 3,5-diglucoside, naringenin-7-O-β-D-glucoside, tannins, catechins, and cyanidin-3-glucoside^24^, betulin, lupeol, lupenone and friedelin^25^

### Anti-oxidant activity

**Figure 2:**
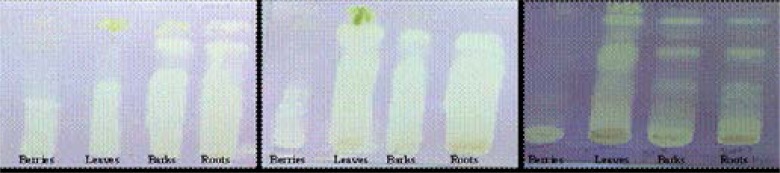
TLC chromatogram of the berries, leaves, bark and roots extracts developed in theBEA system (A) in the EMW system (B) and in the CEF system (C) and sprayed with DPPH.

The qualitative anti-oxidants screening of spraying DPPH on TLC plates indicates the presence of antioxidant compounds in the crude extracts. The anti-oxidant compounds are visualised as yellow spots against the purple background of DPPH, as shown in Figure 2. The number of anti-oxidant compounds identifiable depends on the mobile system used for separation. The Roots extract showed a range of compounds extracted. Most of the antioxidant compounds in this species were polar phenolics compounds, and were better separated with a polar mobile system (CEF).

Quantitative analysis of the scavenging ability showed that acetone extracts exhibited good free radical scavenging activity in a dose-dependent manner. The IC50 values ranged from 250.53 µg/mL to 70.09 µg/mL. Root extract exhibited the highest antioxidant activity (IC_50_ values of 70.09 µg/mL) which was consistent with the activity on TLC-DPPH sprayed plates. However this activity was lower than that of ascorbic acid used as control (IC_50_ value of 9.14 µg/mL). The leaves extract had a high DPPH radical activity compared to the berries and bark. No previous study has been focused on the anti-oxidant activity of *Grewia flava*. However, the anti-oxidant activity of related species is well documented^26^.

### Cytotoxicity

The cytotoxicity was determined using the in vitro assay and tested against Vero monkey kidney cells. The LC_50_ values and the selectivity index calculated are presented in Table 2. The cytotoxicity evaluation is important in the biological activity testing to ensure that the biological activity of the plant extract is not due to a general metabolic toxic effect. In this study, the LC_50_ values ranged from 402.13 to 551.68 µg/mL, while the SI values were between 0.05 and 7.88. The berries extract had the highest LC_50_ (lowest toxicity) of 551.68 µg/mL. The results indicate that the extracts of *Grewia flava* are less toxic to Vero cells at the tested concentration compared to doxorubicin used as control (IC_50_ value of 3.02 µg/mL) and therefore confirm the safety of the use of this edible plant.

**Table 2:** Antioxidant activity (IC_50_ in µg/mL), cytotoxicity against Vero cells (LC_50_ in µg/mL) and selectivity index (SI) of Grewia flava acetone extracts.

			Selectivity index (LC_50_/MIC)			
			
Extracts	IC_50_ (µg/mL)	LC_50_ (µg/mL)	*Bc*	*Ec*	*Sa*	*ST*
Berries	222.34±35.32^a^	551.68±22.55^a^	0.41	4.03	0.50	0.50
Leaves	123.87±26.18^b^	507.21±12.65^b^	7.88	1.00	2.33	1.00
Bark	250.53±43.08^c^	452.69±33.02^c^	1.46	2.07	2.14	0.11
Roots	70.09±17.15^d^	402.13±23.02^d^	5.74	1.00	2.33	0.05
Doxorubicin	nd	3.02±0,03^e^	nd	nd	nd	nd
Vitamin C	9.14±3.24^e^	nd	nd	nd	nd	nd

*BC: Bacillus cereus, EC: Escherichia coli, ST: Staphylococcus aureus, ST: Salmonella typhimurium.* nd = not detected. Data represent the mean ± SD of three independent experiments; values with different letters are significantly different at p< 0.05.

## Conclusion

The indigenous edible plant *Grewia flava* was shown to be non-toxic at the tested concentrations. Root and leaves extracts possess significant antibacterial activity particularly against *Staphylococcus aureus* and therefore could be used to combat bacterial diseases. Further studies have to be conducted to confirm the safety and to isolate the bioactive compound(s).

## References

[1] Bhat RB (2014). Medicinal plants and traditional practices of Xhosa people in the Transkei region of Eastern Cape, South Africa. Indian J Trad Med.

[2] Gurib-Fakim A (2006). Medicinal plants: Traditions of yesterday and drugs of tomorrow. Mol Aspects Med.

[3] Van Wyk BE, van Oudtshoorn B, Gerick N (2009). Medicinal plants of South Africa.

[4] Blum D, Feachem RG (1983). Measuring the Impact of Water Supply and Sanitation Investments on Diarrheal Diseases: Problems of Methodology. Int J Epidemiol.

[5] Sekirov I, Russels SL, Antunes CA, Finlay BB (2010). Gut Microbiota in Health and Disease. Physiol Rev.

[6] Rabe T, van Staden J (1997). Antibacterial activity of South African plants used for medicinal purpose. J Ethnopharmacol.

[7] Tiwara BK, Valdramidis V P, O'Donnell C P, Muthukumarappan K, Bourke P, Cullen P J (2009). Application of Natural Antimicrobials for Food Preservation. J Agric Food Chem.

[8] Chen X-W, Serag ES, Sneed KB, Zhou S-F (2011). Herbal bioactivation, molecular targets and the toxicity relevance. Chem-Biol Interact.

[9] Fennell CW, Lindsey KL, McGaw LJ, Sparg SG, Stafford GI, Elgorashi EE, Grace OM, van Staden J (2004). Assessing African medicinal plants for efficacy and safety : pharmacological screening and toxicology. J Ethnopharmacol.

[10] Ernst E (2004). Risks of herbal medicinal products. Pharmacoepidemiol Drug Saf.

[11] Wali UW, Uddin G, Siddiqui BS (2012). Ethnic uses, Pharmacological and phytochemical profile of genus Grewia. J Asian Nat Prod Res.

[12] Leistner OA (2000). Seed plants of Southern Africa: families and genera. Strelizia.

[13] Bowden BN, Dod B (1978). Tiliaceae, flowering plants of the world.

[14] Curtis BA, Mannheime CA (2005). Tree Atlas of Namibia.

[15] Mainah J (2001). Distribution and association of Grewia flava with other species in the Kalahari environment. Botswana Notes and Records.

[16] Kotze M, Ellof JN (2002). Extraction of antibacterial compounds from Combretum microphyllum (Combrataceae). S Afr J Botany.

[17] Dzoyem JP, McGaw LJ, Eloff JN (2014). In vitro antibacterial, antioxidant and cytotoxic activity of acetone leaf extracts of nine under-investigated Fabaceae tree species leads to potentially useful extracts in animal health and productivity. BMC Complement Altern Med.

[18] Masoko P, Eloff JN (2007). Screening of twenty-four South African combretum and six terminalia species (combretaceae) for antioxidant activities. Afr J Tradit Complement Altern Med.

[19] Brand-Williams W, Cuveleir ME, Berset C (1995). Use of a free radical method to evaluate antioxidant activity. LWTFood Sci Technol.

[20] Eloff JN (1998). Which extractant should be used for the screening and isolation of antimicrobial components from plants?. J Ethnopharmacol.

[21] Zia-U-Haq M, Stanković MS, Rizwan K, Feo VD (2013). Grewia asiatica L., a food plant with multiple uses. Molecules.

[22] Gupta P, Sharma A, Verma AK (2012). GC/MS profiling and antimicrobial effect of six Indian tropical fruit residues against clinically pathogenic bacterial strain. Int J Adv Pharm Res.

[23] Taganna JC, Quanico JP, Perono RMG, Amor EC, Rivera WL (2011). Tannin-rich fraction from Terminalia catappa inhibits quorum sensing (QS) in Chromobacterium violaceum and the QS-controlled biofilm maturation and LasA staphylolytic activity in Pseudomonas aeruginosa. J Ethnopharmacol.

[24] Chattopadhyay S, Pakrashi SC (1975). Indian medicinal plants. XXXIV. Triterpenes from Grewia asiatica. J Ind Chem Sci.

[25] Abou Zeid AHS, Sleem AA (2005). Anti-hyperlipidemic effect and lipoidal constituents of Grewia asiatica L. leaves. Bull Natl Res Cent.

[26] Asghar MN, Khan IU, Sherin L, Ashfaq M (2008). Evaluation of antioxidant activity of Grewia asiatica berry using 2,2-azinobis-(3-ethylbenzoline-6-sulphonic acid) and N,N-dimethyl-p-phenylenediamine radical cations decolourazation assays. Asian J Chem.

